# Mental health integration in primary health services after the earthquake in Nepal: a mixed-methods program evaluation

**DOI:** 10.1017/gmh.2021.8

**Published:** 2021-03-15

**Authors:** Ashley Leichner, Aemal Akhtar, Caoimhe Nic a Bhaird, Rebecca Wener, Shiromi M. Perera, Inka Weissbecker

**Affiliations:** 1 Health Technical Unit, International Rescue Committee, Washington, DC, USA; 2 School of Psychology, University of New South Wales, Sydney, NSW, Australia; 3 MHPSS Consultant, Ireland; 4 Governance and Global Health, Global Communities, Maryland, USA; 5 Technical Unit, International Medical Corps, Washington, DC, USA

**Keywords:** Humanitarian, mental health in primary care, mental health, Nepal, psychosocial support

## Abstract

**Background:**

In the aftermath of the devastating 2015 earthquakes in Nepal, three non-governmental organizations collaborated to develop a program responding to the immediate mental health and psychosocial support (MHPSS) needs in three severely affected districts: Dhading, Gorkha, and Sindhuli. The program was implemented between April 2015 and February 2017 and aimed to (i) strengthen health worker capacity to provide integrated MHPSS services; and (ii) increase access to mental health services. This paper describes the program's implementation and the results of a pragmatic evaluation of the program's overall reach, effectiveness, and lessons learned.

**Methods:**

The mixed-methods evaluation used routine program data, quantitative data from pre- and post-tests conducted with trainees and service users, and qualitative data from stakeholder interviews and focus group discussions.

**Results:**

A total of 1041 health workers received MHPSS training and supervision. Participants demonstrated significant improvements in skills, knowledge, and self-rated perceived competency. Trainees went on to provide MHPSS services to 3422 people. The most commonly identified presenting problems were epilepsy (29%) and depression (26%). A total of 67% of service users reported being ‘completely satisfied’ with the services received and 83% of those experiencing severe functional impairments on enrollment demonstrated improvement after receiving services.

**Conclusions:**

Despite operational challenges, the program successfully engaged both laypeople and health workers to provide MHPSS in the aftermath of the crisis. Lessons learned can inform the planning and implementation of future training and integration programs to provide large-scale MHPSS efforts in humanitarian settings.

## Background

During the spring of 2015, two major earthquakes devastated parts of Nepal, destroying more than half a million houses and leaving 2.8 million people homeless (National Reconstruction Authority, 2016; Sherchan *et al*., 2017). The earthquakes resulted in 8790 deaths and left over 22 000 people injured. Over 1000 health facilities were affected, leaving thousands without access to healthcare (Ministry of Health and Population, 2015).

The World Health Organization (WHO) estimates that rates of mild or moderate mental disorders increase from a baseline of 10% in the general population to 15–20% following emergencies (World Health Organization *et al*., 2012). Multiple risk factors for mental disorders were prevalent in Nepal prior to the earthquake, including poverty, gender and caste inequality (Brenman *et al*., 2014), and exposure to violence during the recent decade-long civil war (Singh *et al*., 2005). A study conducted 4 months after the second earthquake found that 34.2% of people in affected districts met criteria for depression, 20.4% reported hazardous alcohol use, and 10.9% reported suicidal ideation (Kane *et al*., 2017).

### Program activities and objectives

This paper reports on the results of a mental health and psychosocial support (MHPSS) program implemented from April 2015 to February 2017 in three severely affected districts of Nepal: Dhading, Gorkha,^1^ and Sindhuli. The program was a collaboration between *International Medical Corps* (IMC), *Transcultural Psychosocial Organization Nepal* (TPO), *Integrated Community Development Campaign* (ICDC), and the Nepal Ministry of Health. It was designed to respond to the immediate MHPSS needs following the earthquakes and to support longer-term integration of MHPSS services into government primary health care services.

The two main program objectives were:

Objective 1: To strengthen the capacity of health workers and community members to provide high-quality MHPSS services through training and supervision.

Objective 2: To provide community-based MHPSS services

#### Objective 1: strengthen the capacity of health workers and community members to provide high-quality MHPSS services through training and supervision

The program strengthened capacity through a layered support system involving community members, non-governmental organization (NGO) staff, and government health workers with different levels of specialization. The majority of participating health workers had little or no prior experience in providing MHPSS.

**Fig. 1.**
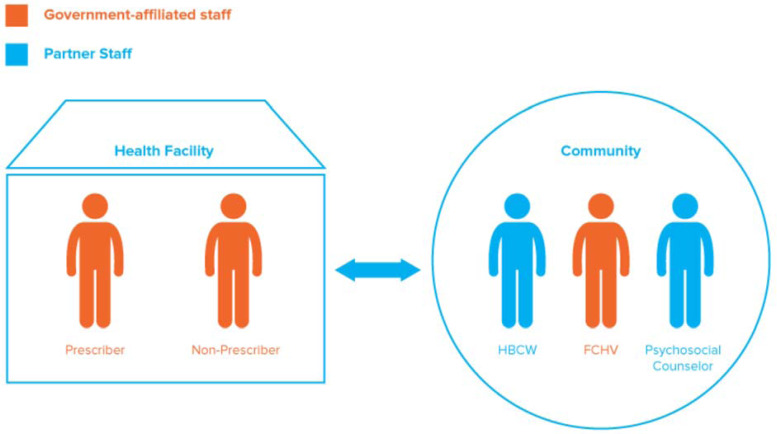
Illustration of the different types of health workers trained as part of the program.

In the initial emergency response phase, community members from 10 severely-affected districts were trained to provide psychological first aid (PFA), an evidence-informed approach providing emotional and practical support to people who recently experienced extremely stressful events (World Health Organization *et al*., 2013). This initial PFA training was rolled out in the immediate aftermath of the earthquakes and pre- and post-test data were not collected at trainings.^2^ While the initial response was ongoing, IMC, TPO, ICDC, and the Ministry of Health collaborated to design a longer-term mental health integration project, which is the focus of this paper. The program trained government health workers and partner NGO staff to provide MHPSS services, focusing on six priority mental health conditions [depression, psychosis, epilepsy, suicide, alcohol use disorders (AUDs), and post-traumatic stress disorder (PTSD)]. The priority conditions were selected based on consultation with national and local stakeholders regarding local prevalence, disease burden, and culturally acceptable treatments. Details of program activities have been published elsewhere (International Medical Corps, 2017). The curricula were based on the WHO Mental Health Gap Action Programme Intervention Guide (mhGAP-IG), the mhGAP Humanitarian Intervention Guide (mhGAP-HIG), (World Health Organization, 2010; World Health Organization, 2015a) and TPO materials. TPO materials included: (1) a *basic psychosocial support training package for home-based care workers* (*HBCWs*), designed to develop community members' skills in basic psychosocial care and appropriate referral; (2) a training package to upskill HBCWs to become psychosocial counselors (PSCs); (3) a *female community health volunteers* (*FCHVs*) *training package* to increase skills in identification and referral of people with mental health and psychosocial problems; and (4) a *mental health orientation for community leaders* to increase their knowledge about available MHPSS services, needs, and referral. Government health workers from 78 health facilities participated, including both ‘prescribers’ (doctors, health assistants, and auxiliary health workers) and ‘non-prescribers’ (staff nurses, auxiliary nurse midwives, and FCHVs). In addition to the participating government health workers, TPO and ICDC hired, trained, and supervised PSCs and HBCWs, community members recruited to provide an intermediate level of care between community support and health facilities. Figure 1 illustrates the health workers trained, showing whether they were government-affiliated or partner staff, and whether they were roving community-workers or based in facilities. Government-affiliated staff were trained with the aim of enabling them to incorporate MHPSS into their regular healthcare duties so that they could continue to provide MHPSS care beyond the duration of the program. Partner staff (PSCs and HBCWs) were hired only for the duration of the program, to support the initial development of the referral network between health facilities and the community. Curricula were tailored to the different groups of trainees according to their roles and responsibilities: prescribers received 9 days of training, non-prescribers received 4 days, and PSCs received 12 days. The training schedule also varied depending on the partner and existing capacities at the different sites, for instance, HBCWs received 5 days in Gorkha and Sindhuli and 20 days in Dhading (Supplement 1). Refresher trainings occurred 6–18 months after the initial training for prescribers, and after 18 months for non-prescribers and PSCs. In addition to training, participants received monthly mentoring and supervision from clinical psychologists and psychiatrists. Supervisors were based in each of the three program districts and were staff of IMC, TPO, or ICDC.

#### Objective 2: provide community-based MHPSS services

Following the training, health workers provided MHPSS services at existing government health facilities and through home-visits. IMC procured psychotropic medication in three batches over the course of the program (November 2015, February 2016, and August 2016) and delivered them to the government health authorities in each of the three districts for distribution among participating health facilities. The range of medications procured was restricted to those listed on the Government of Nepal Free Essential Drug List on the basis that service users would be able to continue to access their prescribed medications after the program ended (Government of Nepal, 2014). The medications included 5 of the 23 medications recommended on the WHO Model List of Essential Medicines for psychotic disorders, depressive disorders, and epilepsy (World Health Organization, 2015*b*). Community education and outreach activities were conducted to raise awareness about the services, to decrease stigma, and to increase access.

## Methods

This paper describes the impact of the program with respect to the two primary objectives, reporting on (1) training outcomes in terms of competency, and knowledge related to mental health service provision, (2) service user outcomes related to the use of program services. A mixed-methods program evaluation was conducted to assess the program's effectiveness in meeting its objectives. The evaluation involved secondary analysis of routine monitoring data collected throughout the program and evaluation data collected at program close in February 2017.

### Measuring trainee knowledge and skills

Socio-demographic data collected from training attendees included: sex, profession, and employment location. Pre- and post-training tests developed by TPO were administered to examine changes in knowledge. Total scores were calculated as percentages.^3^

The IMC *Perceived Competency Checklist* (PCC) is a 13-item questionnaire with which participants rate their own competency on a range of skills using a five-point Likert scale. The general competency subscale consists of four items, assessing ethical practice, supportive communication, understanding stress, and stigma. The clinical competency subscale consists of nine items on assessment, diagnosis, intervention, referral, and documentation. Scores for each subscale are calculated by summing the ratings and then dividing by the overall number of questions. The PCC was administered on the first day of training and again on the last day of refresher training.

The *Assessment of Clinical Expertise* (*ACE*) and the *ENhancing Assessment of Common Therapeutic factors* (*ENACT-18*) are questionnaires used by expert supervisors (psychiatrists) to rate each trainee's competence while observing a clinical encounter between the trainee and a service user. The ACE, an adapted version of the mini-clinical evaluation exercise originally developed by the Royal College of Psychiatrists (Norcini, 2005), is used to evaluate a clinician's expertise on 10 items using a scale of 1–6. The ACE checklist was first administered within 3 months of training, and again during the final supervision session or the final day of refresher training, with a maximum period of 18 months between assessments. The ACE was used to assess the abilities of the clinical trainees (prescribers, non-prescribers, and PSCs), including clinical assessment, care planning, and record keeping (Supplement 2). The ENACT-18, which has previously been validated in Nepali (Kohrt *et al*., 2015), consists of 18 items rated on a scale of 1–3 to assess participant competence during the last day of training and again during the last day of refresher training, The final score is the mean score across all items. The ENACT was used in conjunction with the ACE to evaluate verbal and non-verbal communication (Supplement 2).

### Measuring service user outcomes

Data were collected from all service users who received MHPSS services at the 78 participating health facilities and those who were reached by FCHVs/HBCWs during home visits. Service users in the community were detected using the Community Informant Detection Tool (CIDT) (Jordans *et al*., 2017). None of the participating facilities documented mental disorders in clinical charts prior to program implementation. The program introduced two documentation procedures: an outpatient mental health register at each facility, and an outpatient docket retained by service users (on which details of each clinical encounter were documented). Clinical encounters were defined as an appointment between the participant and health services (i.e. HBCW, CHW, PSC, prescriber, and non-prescriber). The following information was recorded for all service users: sex, age, and area of residence, date of the first contact with a health provider, presenting problem, and whether the medication was prescribed during an initial visit or during follow-up. Presenting problem was documented by the service provider at the service user's first contact with services. It was therefore based on the service provider's initial assessment using knowledge gained through their mhGAP training, rather than a formal diagnosis by a health professional with specialist psychiatric training. While these staff were receiving supervision from qualified clinicians, results relating to presenting problem must be interpreted with caution as they do not represent formal psychiatric diagnoses.

*Service user functioning* and *satisfaction with services* were assessed for a subset of service users. To reduce the risk of social desirability bias, functioning and satisfaction scales needed to be administered by an independent staff member, rather than by the service user's primary care provider. Due to the limited number of staff and the need to balance their service provision and data-collection responsibilities, it was not feasible to administer these scales to all service users. These measures were therefore administered to service users receiving medication under the care of a prescriber because it was feasible for an independent HBCW to administer them. General health and functioning were assessed using the WHO Disability Assessment Schedule II (WHO-DAS II) (World Health Organization, 2001) 3 and 9 months after initial medication prescription. The WHO-DAS II, previously validated in Nepali, consists of 12 interviewer-administered questions spanning six domains (communication, mobility, self-care, interpersonal, life activities, and participation) with responses scored on a five-point scale of difficulty (none, mild, moderate, severe, or extreme). Satisfaction with services received was assessed 9 months after initial medication prescription by asking service users whether they were ‘completely satisfied’, ‘somewhat satisfied’ or ‘not satisfied’.

### Quantitative data analysis

A within-sample one-sided *t* test (with *α* = 0.05) was conducted to compare the pre- and post-training scores for the knowledge tests, PCC, ACE, and ENACT-18 assessments. A within-sample one-sided *t* test was used to assess changes in service user functioning between their first recorded WHO-DAS II score at 3 months following their first prescription and their final score at 9 months. Changes were compared by socio-demographic characteristics and by the initial presenting problem. The mean difference, the 95% confidence interval (CI), and the *p* value are reported. One-sided *t* tests were used based on the assumption that there would be improvement over the course of trainings and treatment. All quantitative analyses were conducted in SAS 9.4^©^ (SAS Institute, Cary, North Carolina).

### Qualitative data analysis

A thematic framework analysis (Supplement 3) was conducted using MAXQDA (Verbi Software, Berlin, Germany) to identify the primary themes associated with each program objective (Braun and Clarke, 2006). This paper presents qualitative findings that elucidate the quantitative results, namely those relating to the impact of the MHPSS trainings and service provision on community members, and areas for program improvement highlighted by participants.

## Results

### Objective 1. capacity building

A total of 1041 health workers from 78 health facilities participated in the MHPSS trainings (Table 1). Majority were prescribing staff (*N* = 221, 50.8%). PSCs represented the lowest proportion (4.14%).

**Table 1:** Descriptive data for training attendees by profession

	Profession				
Variable	HBCW (*N* = 66)	PSC (*N* = 18)	Non-Prescriber (*N* = 130)	Prescriber (*N* = 221)	Total (*N* = 435)
Sex					
Female (%)	48 (72.73)	8 (44.44)	94 (72.31)	59 (26.7)	209 (48.05)
Male (%)	18 (27.27)	10 (55.56)	36 (27.69)	162 (73.3)	226 (51.95)
Training days					
Mean (s.d.)	15.82 (10.59)	10.61 (2.68)	4.99 (0.09)	8.88 (0.69)	-
Median (IQR)	20 (10,20)	12 (12,12)	5 (5,5)	9 (9,9)	-
Number supervision visits					
Mean (s.d.)	6.09 (8.42)	11.72 (2.16)	5.35 (2.9)	3.81 (2.28)	4.91 (4.47)
Median (IQR)	3 (2,5)	13 (11,13)	6 (3,7)	4 (2,6)	5 (2,6)

Acronyms: IRD – Interquartile range s.d. – standard deviation.

A total of 435 government health workers and NGO staff members received MHPSS trainings over the course of the program. Statistically significant improvements were found between mean pre- and post-training scores on all tests (*p* < 0.0001): 85% demonstrated improvements in knowledge tests; 92% and 97% demonstrated improvements in perceived general and clinical competency, respectively; 82% improved in their ACE scores; and 75% improved in their ENACT-18 scores. Table 2 presents the scores by profession for each test. HBCWs did not complete the PCC, ENACT-18, or ACE tests. Within each trainee group, all post-test scores were significantly greater than the corresponding pre-test scores, except for PCC scores for PSCs, where the improvement between pre- and post-training was not statistically significant.^4^

**Table 2:** Training outcome tool results by profession

Knowledge test				
	Pre-training score *N* Mean (s.d.)	Post-training score *N* Mean (s.d.)	Difference^a^ *N* Mean (95% CI)	*p* value
HBCW	43 71.33 (14.14)	43 88.66 (10.27)	43 17.33 (12.68,21.98)	<0.0001
PSC	18 58.65 (8.85)	18 72.66 (11.32)	18 14.01 (8.69,19.32)	<0.0001
Non-Prescriber	130 60.97 (14.68)	127 73.57 (12.99)	127 12.46 (10.02,14.9)	<0.0001
Prescriber	200 67.34 (11.38)	198 79.2 (9.94)	197 11.94 (10.49,13.38)	<0.0001
**Perceived general competency scores**				
	**Pre-training score *N* Mean (s.d.)**	**Post-training score *N* Mean (s.d.)**	**Difference**^a^ ***N* Mean (95% CI)**	***p* value**
PSC	18 3.06 (0.77)	13 3.17 (0.55)	13 0.19 (−0.14, 0.52)	0.1599
Non-Prescriber	93 2.89 (0.82)	48 3.5(0.71)	48 0.58 (0.38, 0.81)	<0.001
Prescriber	202 3.07 (0.69)	162 3.95 (0.79)	162 0.88 (0.76, 1)	<0.001
**Perceived clinical competency scores**				
	**Pre-training score *N* Mean (s.d.)**	**Post-training score *N* Mean (s.d.)**	**Difference**^a^ ***N* Mean (95% CI)**	***p* value**
PSC	18 2.78 (1.02)	13 3.41 (0.55)	13 0.44 (0.11, 0.77)	0.1599
Non-Prescriber	93 2.34 (0.78)	35 2.94 (0.81)	35 0.37 (0.37, 0.37)	<0.0001
Prescriber	202 2.7 (0.86)	162 4.09 (0.62)	162 1.41 (1.27, 1.55)	<0.0001
**ENACT-18 scores**				
	**Pre-training score *N* Mean (s.d.)**	**Post-training score *N* Mean (s.d.)**	**Difference**^a^ ***N* Mean (95% CI)**	***p* value**
PSC	18 2.03 (0.44)	13 2.75 (0.19)	13 0.65 (0.34, 0.97)	0.0003
Non-Prescriber	128 1.87 (0.36)	79 2.31 (0.33)	78 0.46 (0.37, 0.56)	<0.0001
Prescriber	201 2.13 (0.42)	170 2.27 (0.39)	170 0.12 (0.07, 0.18)	<0.0001
**ACE scores**				
	**Pre-training score *N* Mean (s.d.)**	**Post-training score *N* Mean (s.d.)**	**Difference**^a^ ***N* Mean (95% CI)**	***p* value**
PSC	18 45.66 (10.59)	16 57.42 (7.55)	16 13.64 (8.47, 18.8)	<0.0001
Non-Prescriber	69 42.69 (9.73)	62 50.43 (11.97)	62 (6.97, 10.37)	<0.0001
Prescriber	180 53.71 (13.61)	180 62.94 (7.07)	167 8.53 (6.35, 10.72)	<0.0001

Acronyms: CI – confidence interval, s.d. – standard deviation.

a Difference only includes participants that had both pre- and post-scores for the outcome measures.

Over the course of the program, health workers received a mean of five supervision sessions (face-to-face or by telephone), with PSCs receiving the highest number of supervision sessions (mean = 12).

In focus group discussions (FGDs) and interviews, health workers commonly reported that the trainings had led to enhanced knowledge, increased confidence in the detection, and diagnosis of mental illness, and improved quality of interactions with service users presenting with mental health problems (Panel A). The supervision and refresher trainings were considered a key strength of the program.

**Table:** 

**Panel A – Illustrative qualitative data**
‘Our confidence level has increased. Before, even for depression, if we had to prescribe a dose, we would have to think twice. But now it's really easy to prescribe medication because we understand the side effects. The patients have improved and our confidence has increased.’ (Prescriber, Dhading, FGD)
‘Previously, I found it difficult to deal with mental health patients, but now this program is helping me a lot in treating and diagnosing mental health disorders. I used to be worried when a patient with psychosis came for treatment, but now I know how to deal with it and treat it.’ (Prescriber, Dhading, FGD)
‘We learned how to communicate properly, about talking styles, about how to treat service users and how to understand their needs.’ (HBCW, Gorkha, interview)

Interview and FGD participants were asked to share the challenges they faced and suggest areas for improvement in future trainings. Suggestions included covering a greater number of disorders in the trainings and simplifying training materials. Trainees also said that they would have liked a greater focus on counseling techniques. The supervision sessions were considered to be extremely useful, but there was a desire for supervision to be more regular and to be accompanied by more frequent refresher trainings.

A full list of themes and sub-themes relating to the trainings is provided in Supplement 3.

### Objective 2. service provision

Over the course of the program, MHPSS services were provided to 3422 service users (42% male and 58% female) through community health facilities and home visits (Table 3). The majority of beneficiaries were in the Dhading district (42%). The mean age of service users was 36.32 (s.d. = 16.34), with the oldest service users in Gorkha (40.28, s.d. = 15.7).

**Table 3:** Descriptive data for service users by presenting problems

	Presenting problem							
Variable	AUD (*n* = 443)	Anxiety (*n* = 143)	Depression (*n* = 760)	Epilepsy (*n* = 885)	PTSD (*n* = 82)	Psychosis (*n* = 601)	Other (*n* = 508)	Total (*N* = 3422)
Age								
Mean (s.d.)	42.89 (13.91)	35.64 (15.34)	39.84 (15.73)	31.98 (16.38)	38.16 (16.07)	39.15 (15.76)	29.43 (15.75)	36.32 (16.34)
Median (IQR)	42 (35, 52)	35 (22, 46)	39 (28.5, 50)	30 (19, 43)	35.5 (26, 45)	38 (28, 50)	26 (16, 40)	35 (23, 48)
Categorical *N* (%)								
<18	16 (3.61)	17 (11.89)	54 (7.11)	192 (21.69)	6 (7.32)	35 (5.82)	145 (28.54)	465 (13.59)
18–24	26 (5.87)	26 (18.18)	83 (10.92)	160 (18.08)	11 (13.41)	81 (13.48)	89 (17.52)	476 (13.91)
25–59	342 (77.20)	89 (62.24)	532 (70)	481 (54.35)	54 (65.85)	414 (68.89)	246 (48.43)	2158 (63.06)
60 +	59 (13.32)	11 (7.69)	91 (11.97)	52 (5.88)	11 (13.41)	71 (11.81)	28 (5.51)	323 (9.44)
Gender								
Female (%)	128 (29.89)	100 (69.93)	537 (70.66)	442 (49.94)	52 (63.41)	335 (55.74)	384 (75.59)	1978 (57.8)
Male (%)	315 (71.11)	43 (30.07)	223 (29.34)	443 (50.06)	30 (36.59)	266 (44.26)	124 (24.41)	1444 (42.2)
Number of contact								
Mean (s.d.)	3.4 (3.94)	2.28 (2.84)	2.91 (3.96)	4.49 (5.03)	2.8 (2.56)	3.58 (4.0)	2.75 (2.71)	3.4 (3.94)
Median (IQR)	2 (1,4)	1 (1,2)	2 (1,3)	2 (1,5)	2 (1, 4)	2 (1, 4)	2 (1,3)	2 (1,4)
First contact								
HBCW *n*(%)	32 (7.22)	1 (0.7)	29 (3.82)	32 (3.62)	5 (6.1)	36 (5.99)	79 (15.55)	214 (6.25)
FCHV *n*(%)	220 (49.66)	2 (1.4)	132 (17.37)	204 (23.05)	17 (20.73)	172 (28.62)	62 (12.2)	809 (23.64)
PSC *n*(%)	46 (10.38)	28 (19.58)	131 (17.24)	141 (15.93)	23 (28.05)	109 (18.14)	152 (29.92)	630 (18.41)
Non-Prescriber *n*(%)	46 (10.38)	41 (28.67)	82 (10.79)	80 (9.04)	14 (17.07)	35 (5.82)	77 (15.16)	375 (10.96)
Prescriber *n*(%)	82 (18.51)	62 (43.36)	352 (46.32)	373 (42.15)	19 (23.17)	217 (36.11)	120 (23.62)	1225 (35.8)
Unspecified *n*(%)	17 (3.84)	9 (6.29)	34 (4.47)	55 (6.21)	4 (4.88)	32 (5.32)	18 (3.54)	169 (4.94)
Type of counseling								
Individual *n*(%)	127 (28.67)	53 (37.06)	296 (38.95)	354 (40)	41 (50)	246 (40.93)	243 (47.83)	1360 (39.74)
Family *n*(%)	43 (9.71)	4 (2.8)	43 (5.66)	76 (8.59)	5 (6.1)	79 (13.14)	42 (8.27)	292 (8.53)
Medication *N* (%)	88 (19.86)	65 (45.45)	368 (48.42)	519 (58.64)	20 (24.39)	257 (42.76)	107 (21.06)	1424 (41.61)

Acronyms: IQR – interquartile range, s.d. – standard deviation.

The most common presenting problems were epilepsy (28%), depression (22%), psychosis (18%), and AUD (13%). Over the course of the program, 438 service users (12.8%) presented with more than one problem. A total of 19% of service users presented with problems other than the six pre-defined categories, including conversion disorder, suicidal ideation, and psychosocial problems. More than a quarter of ‘other’ presenting problems were reported by children and adolescents.

The medication was provided to 1424 (42%) service users. Those presenting with epilepsy received medication most frequently (59%), followed by those presenting with depression, anxiety, and psychosis (42%–48%). Prior to the program, some participants were already receiving psychotropic medication from other sources (data on the specific number was not available). The qualitative data revealed that some service users preferred to continue to receive medication from their usual source while participating in the program for additional support, such as counseling.

Over the course of the program (22 months), the mean number of service contacts per service user was 3.4 and was greatest for those presenting with epilepsy (4.49) and lowest for those presenting with anxiety disorders (2.28). Service users presenting with anxiety, depression, epilepsy, and psychosis typically made their first contact with prescribers. FCHVs and PSCs were the second and third most common healthcare professionals, respectively, that served as entry points into MHPSS services across all disorders.

### Service user outcomes

A total of 476 (13.91%) service users completed the WHO-DAS II assessment of functioning and reported their levels of satisfaction with the services provided. Additional socio-demographic and clinical measures are reported in Table 4.

**Table 4:** Descriptive data for service users who completed outcome measures

	Region			
Variable	Dhading (*N* = 129)	Gorkha (*N* = 157)	Sindhuli (*N* = 190)	Overall (*N* = 476)
Age				
Mean (95% CI)	34.2 (18.48)	43.17 (15.71)	34.02 (15.57)	37.11 (16.94)
Median (IQR)	29 (20, 47)	43 (33, 53)	33.5 (22, 43)	35.5 (24, 50)
Gender				
Female (%)	63 (48.84)	84 (53.5)	97 (51.05)	244 (51.26)
Male (%)	66 (51.16)	73 (46.5)	93 (48.95)	232 (48.74)
Education				
Illiterate	47 (36.43)	35 (22.29)	45 (23.68)	127 (26.68)
Literate	82 (63.57)	122 (77.71)	145 (76.32)	349 (73.32)
Services received				
Medication only	0 (0)	73 (46.5)	95 (50)	168 (35.29)
Medication and counseling services	129 (100)	84 (53.5)	95 (50)	308 (64.71)
Presenting problem; *N* (%)				
Epilepsy	39 (30.23)	32 (20.38)	84 (44.21)	155 (32.56)
Depression	34 (26.36)	52 (33.12)	22 (11.58)	108 (22.69)
Psychosis	12 (9.30)	25 (15.92)	28 (14.74)	65 (13.66)
Others	36 (27.91)	4 (2.55)	13 (6.84)	53 (11.13)
Missing	0 (0)	25 (15.92)	27 (14.21)	52 (10.92)
AUD	7 (5.43)	13 (8.28)	2 (1.05)	22 (4.62)
Anxiety	0 (0)	4 (2.55)	10 (5.26)	14 (2.94)
PTSD	1 (0.78)	2 (1.27)	4 (2.11)	7 (1.47)

For 52 service users who completed the outcome measures (10.92%), it was not possible to link their outcomes to their ‘presenting problems’ due to missing data.^5^ Among service users who completed the measures, epilepsy was the most common presenting problem (32.56%). A total of 306 (64.71%) of those who completed assessments received both medical and counseling services, while the remaining 170 (35.29%) received only medication. The WHO-DAS II was used to evaluate overall improvement in functioning over the course of the program. A total of 55.7% of service users showed improvements, with significant improvements made by those attending health services in Dhading (−8.66, *p* < 0.0001) and Sindhuli (−4.51, *p* = 0.026), but not in Gorkha (1.88, *p* = 0.766). Service users with the following characteristics were more likely to demonstrate significant improvements in their functioning 9 months after receiving an initial prescription (Table 5): those aged 25–29 years (−2.88, *p* = 0.0428), males (−3.76, *p* = 0.0269), those who were literate (−5.1, *p* = 0.0006), those presenting with anxiety problems (−14.57, *p* = 0.0368), and those with problems classified as ‘other’ (−9.1, *p* = 0.0060). Service users were significantly less likely to improve if they were illiterate (1.84) or presenting with depression (0.52). An analysis of the WHODAS II subscales showed significant improvements in social functioning, mobility, self-care, and the number of days in which they were unable to carry out their ‘usual activities or work’. A total of 89% of service users with severe functional impairments, defined as scores above 75 on the WHODAS II, improved over the course of the program.

**Table 5:** WHO-DAS II results for service users

	WHODAS score 1 *N* Mean (s.d.)	WHODAS score 2 *N* Mean (s.d.)	Difference score *N* Mean (95% CI)	*p* value
Age				
<18	70 37.34 (19.92)	64 35.42 (19)	64 −4.02 (−9.39, 1.35)	0.0697
18–24	60 39.8 (23.12)	56 37.98 (21.97)	56 −2.98 (−11.74, 5.78)	0.2492
25–59	293 39.77(22.56)	285 37.2 (21.66)	285 −2.88 (−6.17, 0.41)	0.0428
60 +	53 47.09 (22.83)	51 42.65 (27.3)	51 −5.11 (−15.14, 4.9)	0.1549
Gender				
Female	244 41.56 (22.02)	232 39.51 (23.48)	232 −2.86 (−6.57, 0.84)	0.0646
Male	232 38.84 (22.7)	224 35.73 (20.36)	224 −3.76 (−7.58, 0.06)	0.0269
Literacy				
Illiterate	127 36.53 (22.97)	118 39.41 (23.15)	118 1.84 (−3.43, 7.11)	0.7545
Literate	349 41.58 (22.03)	338 37.04 (21.67)	338 −5.1 (−8.16, −2.04)	0.0006
Presenting problem				
AUD	22 43.14 (24.93)	22 38.16 (26.02)	22 −4.97 (−18.14, 8.19)	0.2205
Anxiety	14 48.64 (21.46)	14 34.08 (19.98)	14 −14.57 (−30.73, 1.6)	0.0368
Depression	108 40.76 (20.54)	103 41.44 (22.71)	103 0.52 (−4.98, 6.02)	0.5745
Epilepsy	155 36.92 (22.38)	147 35.74 (21.71)	147 −2.6 (−7.15, 1.95)	0.1305
PTSD	7 52.57 (24.76)	7 34.52 (12.2)	7 −18.05 (−42.69, 6.6)	0.0617
Psychosis	65 45.08 (26.26)	64 41.24 (20.31)	64 −4.48 (−12.65, 3.7)	0.1390
Others	53 37.77 (19.31)	47 29.3 (22.4)	47 −9.1 (−16.11, −2.1)	0.0060

Overall, 67% of service users reported being ‘completely satisfied’ with the services they had received, 31% reported being ‘somewhat satisfied’, and 2% reported being ‘unsatisfied’. FGDs and interviews corroborated the finding that services users were generally very satisfied with the services provided. Many participants reported a newfound sense of hope that their condition was treatable (Panel B).

**Table:** 

**Panel B – Illustrative qualitative data**
‘I feel that everything has changed. I have been transformed … It's all improved because of counseling.’ (Service User, Dhading, FGD)
‘I used to feel like I didn't have strength to do things. Things that I wanted to do seemed impossible and I would often get sad. But now if I start something I feel I have the strength to complete it.’ (Service User, Sindhuli, FGD)
‘There was nothing we didn't do to try and treat my daughter-in-law. We took her to everywhere possible, even to India. I faced lots of problems personally due to that. But now, after your service, we are really happy.’ (Carer, Sindhuli, FGD)
‘There are people who have not been able to get a diagnosis for 12 or 13 years. They had visited different places for treatment but no treatment cured it. But the medication from our program has cured those patients. They want this program to last longer.’ (HBCW, Gorkha, interview)

During the FGDs and interviews, several common themes emerged about the impact of service provision. Many participants reported that the program improved their mental health by having a lasting impact on multiple facets of their lives, such as relationships with friends and family, daily functioning, and their livelihood and finances. Health workers reported feeling a greater sense of satisfaction with their work, and felt that being able to provide better care was having a positive effect on their own personal lives.

When asked about remaining challenges and areas for improvement, health workers reported that certain staff roles were underutilized, especially government roles, and that frequent staff rotations during program implementation resulted in the loss of trained staff from participating facilities. Participants suggested that it would be better to focus on training a greater number of government staff, rather than spending resources on shorter-term NGO staff. Service providers also recommended increasing the involvement of the community and spiritual leaders in developing pathways to care, to improve the reach and impact of the program. Finally, a commonly reported challenge was that, once IMC had supplied medications to the district hospital in a region, there were often delays in distribution to the primary health care centers. This was identified as a source of frustration among primary health staff and service users, who reported having to wait for medication or seek treatment elsewhere. Notwithstanding these challenges at the local level, the program had a broader positive impact on medication supply nationally. Through participation in national strategy meetings, program staff contributed to three major changes in mental health policy: authorization for local authorities to procure psychotropic medication; revisions to the Government of Nepal Free Essential Drug List to include modern antipsychotics and Selective Serotonin Reuptake Inhibitors (SSRIs); and updates to the national Standard Treatment Protocol for primary care mental health services.

## Discussion

Significant improvements in MHPSS knowledge, skills, and perceived competencies among health workers were observed over the course of trainings and supervisions. Trainees reported that their abilities to support people with mental health problems was greatly enhanced, and described having improved confidence in the detection, diagnosis, and treatment of mental illness. They emphasized that their understanding of mental health and illness prior to the program had been extremely limited. Additional benefits included the establishment of MHPSS services in Gorkha, where there had been no integrated services previously, and initiating bulk procurement of psychotropic medications in districts where these medicines had not previously been available. Overall key programmatic lessons learned included: establishing a manageable set of focused indicators with clearly defined targets early in the program; conducting baseline assessments before interventions whenever possible; and training program and partner staff on the routine analysis of program data to investigate trends and adapt programs accordingly.

### Capacity building

Trainees identified the ongoing supervision and refresher trainings as key program strengths that were central to their ability to provide high-quality MHPSS services. This highlights the importance of prioritizing the development of robust long-term supervision structures when designing and implementing future programs.

The service model of engaging existing health workers whilst training additional psychosocial workers to provide community-based care was based on the previous work of TPO (the PRIME project) (Jordans *et al*., 2015). In this model, some NGO staff (PSCs and HBCWs) were hired for the duration of the project only, as a temporary support to government staff. While these roles were not sustainable after program closure (as their salaries would no longer be funded through the project), evaluation participants nonetheless considered that the roles had been a valuable supplement to government services during the early stages of program development. Some PSCs and HBCWs highlighted that they intended to continue to use the knowledge they had gained to support their communities informally after the program ended.

Government-affiliated staff trained as part of the program (prescribers, non-prescribers, and FCHVs) were expected to continue in their roles after the program ended, and to continue to apply what they had learned in their MHPSS training. The extent to which this will sustain in practice without ongoing technical supervision and support is uncertain. A review of the overall MHPSS response to the Nepal earthquakes questioned the sustainability of MHPSS programs initiated by international NGOs, given the lack of government funding and the low capacity of district authorities to maintain interventions (Sherchan *et al*., 2017). Future programs may benefit from dedicating more time during training and supervision sessions early in the program to establishing clear expectations among government health practitioners about the continuation of MHPSS services after program close and the collaborative development of strategies to enhance sustainability (e.g. by securing long-term funds for ongoing expert or peer-supervision). Expert guidance recommends that program managers innovate mechanisms to invest funds for long-term use, for example, by collaborating with the government to seek out opportunities for public-private partnerships (Patel *et al*., 2011). This may be particularly relevant in a context such as Nepal, where public spending on mental health has traditionally been very low (with less than 3% of the national budget spent on health, and less than 1% of this allocated to mental health) (IASC, 2015).

### Service provision, service user outcomes

Both service users and health workers reported that the program led to recovery, improved wellbeing, and a new sense of hope that mental illnesses are treatable within the community. This was supported by both quantitative and qualitative results showing significant improvements in daily functioning and social interaction among service users.

Interestingly, 30% of service users in our sample (*n* = 1023) were identified by HBCWs and FCHVs in community settings, including the majority of AUDs (57%). A previous study in Nepal showed that only 5.1% of people who met the criteria for AUD and 8.1% who had depression had sought any form of formal treatment in the previous year (Luitel *et al*., 2015) which highlights the importance of community outreach. The CIDT used by FCHVs in this study was developed and trialed in Nepal to assist in community identification of AUD, depression, psychosis, and epilepsy (Jordans *et al*., 2017; Subba *et al*., 2017). This tool could be used more broadly to help to improve the reach of mental health services and could assist in identifying persons requiring support in the community.

A comparison of service use across the districts revealed variations in presenting problems and service user satisfaction, which warrant closer monitoring and investigation. For example, in Gorkha, one of the most severely affected regions, while service users demonstrated significant improvements in daily functioning and social interaction overall, improvements were not statistically significant. This highlights the importance of planning for and building data systems that allow for the collection and analysis of program data on a regular basis throughout the program so that such variations can be investigated and addressed in a timely manner.

The rates with which certain disorders presented at the clinics in this program differed from global prevalence estimates, which suggest that depression, followed by anxiety, and AUDs would be the most prevalent disorders (James *et al*., 2018; Thapa *et al*., 2018). Epilepsy was the most common presenting problem, followed by depression, and psychosis. Other researchers have also identified high rates of epilepsy following emergencies (Jones *et al*., 2009; Kane *et al*., 2014). This may reflect the fact that epilepsy and psychotic disorders are often associated with overt externalizing symptoms that may be easier for general healthcare providers to identify in comparison to common mental illnesses such as depression, which can have less obvious symptoms (Mbeya *et al*., 2018). The proportion of service users presenting with depression was particularly high in Gorkha, compared to Dhading and Sindhuli, which may reflect the fact that it was more severely affected by the earthquakes (National Planning Commission, 2015). Kane and colleagues (2017) also found that rates of depression were higher in more severely affected districts. The higher frequencies of AUDs reported in Gorkha and Dhading may reflect differences in the cultural and sociodemographic profile of service users in the different districts. Kane and colleagues found substantially higher rates of alcohol disorder among those identifying as belonging to the more marginalized Janajati and Dalit ethnic groups compared with those identifying as Brahmin or Chhetri (groups in which alcohol consumption has traditionally been considered forbidden) (Kane *et al*., 2017). Collecting systematic socioeconomic data on service users would aid in the interpretation of such variations in future programs.

Persons presenting to the health facilities with anxiety was markedly low in this study relative to global prevalence estimates (Global Burden of Disease Study, 2015; James *et al*., 2018) and studies conducted in Nepal after the earthquakes (Kane *et al*., 2017) – only 4.41% of service users presented with anxiety, the majority of whom were in Sindhuli. Lower rates of anxiety were reported in regions with higher rates of AUD. One possible explanation for this pattern is the self-medication theory: that people use alcohol as a form of self-medication, resulting in high rates of AUD which mask underlying anxiety disorders (Smith and Randall, 2012). Globally, anxiety is considered one of the most common mental disorders (James *et al*., 2018), so it is perhaps then more likely that anxiety was not accurately identified by staff, while disorders with more observable behavioral disturbances (such as epilepsy and psychosis) were more likely to be recognized and to present at health services. As anxiety disorders (other than PTSD) were not among the six conditions prioritized in trainings for this program, staff may not have had sufficient capacity to identify them. It is also possible that, given the high levels of comorbidity between anxiety and depression, cases presenting with comorbid anxiety were categorized as depression (Risal *et al*., 2016; Adewuya *et al*., 2018). The risk of inaccurate diagnoses has been highlighted as a potential threat to quality in evaluations of similar programs in Nepal (Luitel *et al*., 2015; Jordans *et al*., 2016).

During the course of the program, there were reports of medications not being promptly distributed from district hospitals to the primary health clinics in accordance with demand. Future programs may benefit from closer collaboration and training with local health authorities, pharmacists, and store managers to improve supply chain management. This would likely reduce delays in treatment and increase levels of service user satisfaction. This program demonstrated the potential value of advocacy efforts at the local and national levels, with program staff contributing to major changes in mental health policy relating to the procurement of psychotropic medication and national mental health treatment protocols.

### Integration with government services

Best practice guidelines recommend the integration of MHPSS services within primary health care to improve access, decrease the burden on specialized services, and support the transition from emergency response to sustainable development (International Medical Corps, 2018). There has been a growing recognition of a global ‘treatment gap’ in MHPSS, with 76–85% of those in need not having access or receiving treatment (Demyttenaere *et al*., 2004; Kohn *et al*., 2004). International best practice guidelines emphasize the importance of building MHPSS capacity for general health care providers in low- and middle-income countries (Patel *et al*., 2011; World Health Organization, 2013) with a focus on strengthening their knowledge of mental illness, available treatments and supports using training programs such as the WHO mhGAP packages for health workers (World Health Organization, 2010; World Health Organization, 2015*a*) and scalable psychological interventions for lay-workers (Murray *et al*., 2011; World Health Organization, 2018*b*).

The extent to which the information collected by the two documentation procedures introduced as part of this program (outpatient register and docket) was incorporated into government health information systems following the program remains unclear. Closer engagement with service managers to develop sustainable integrated mental health information systems and placing more emphasis on clinical documentation in future training may improve effectiveness and the long-term impact of similar programs (International Medical Corps, 2018; World Health Organization, 2018*a*).

The program supported both short-term and long-term government priorities on strengthening human resources for mental health and improving referral mechanisms. Program activities aligned with Nepal's Post-Disaster Recovery Framework, which listed the MHPSS needs of earthquake-affected populations as a key priority (National Reconstruction Authority, 2016). The program was also consistent with the 1996 National Mental Health Policy of Nepal, which promotes the integration of mental health services into the general health system and training of all government health workers in mental health (Government of Nepal, 1996). The three program partner organizations, along with other stakeholders, were successful in lobbying the government to add a wider range of the modern medications recommended by the WHO to the Government of Nepal Free Essential Drug List (Government of Nepal *et al*., 2016).

Some health workers suggested that certain government roles were under-utilized and that channeling resources for government staff would be more effective than hiring short-term NGO staff. This is consistent with international best practice guidance promoting engagement of existing health service staff, for integration and sustainability (Patel *et al*., 2011). Non-prescribers and FCHVs were the two professional groups reported to be underutilized, with participants attributing this to existing professional hierarchies and a lack of adequate leadership and management encouraging collaboration amongst all professional groups within the health system. However, government staff have a wide variety of commitments other than MHPSS programs and have limited time to dedicate to training and service-provision, particularly in the aftermath of a disaster which caused widespread physical injuries as well as mental distress. When engaging government staff, program planners must consider the burden imposed by additional duties (Patel *et al*., 2011). Developing a phased approach which is responsive to the changing healthcare needs and duties of service providers after an emergency may improve the effectiveness of similar programs.

### Sustainability

Several factors offer cause for optimism regarding sustained program benefits. Large number of people were reached through community outreach activities which provided accurate information on mental health and challenged stigma and misconceptions. There was evidence that the program increased the demand for quality mental health services among both community members and government staff, and developed a momentum for change. With input from IMC, TPO, ICDC, and other NGOs, substantial progress has been made in revising national policies on standard treatment protocols for mental health care and the supply of modern psychotropic medications. These changes may signify an increase in political will and public interest in mental health which, with continued advocacy from local NGOs, can be channeled for longer-term mental health systems strengthening.

### Limitations

The practicalities of collecting data in an evolving emergency setting along with resource constraints led to a number of limitations in this program evaluation which should be considered when interpreting the findings. We were only able to analyze data on ‘presenting problems’ documented by frontline staff (e.g. HBCWs) when service users first presented to services – not formal diagnoses made by specialized providers, which limits their interpretive utility. It was not possible to validate the presenting problems recorded through clinical examinations as this data was not updated after subsequent visits to clinical staff. Additionally, small sample sizes among certain groups led to reduced statistical power when comparing outcomes, making it less likely that changes would be detected in significance tests.

Future programs would benefit from analyzing data throughout the program to pick up any unexpected trends and allow for further investigation. Secondly, resource and time constraints also limited us to collecting service user outcomes for only a subset of service users who were prescribed medications. Outcome results therefore may not be representative of the entire service user population. Although we captured changes in daily functioning in this subsample, changes in functioning among those who received counseling without medication were not captured. Thirdly, data on presenting problems were missing for 10.3% of service users. The loss of data was consistent between the three districts, so it is unlikely that this resulted in a systematic bias.

## Conclusion

This program demonstrated the feasibility and effectiveness of training health staff and lay workers to provide MHPSS services in an emergency setting, with the aim of longer-term health system strengthening. Future programs would benefit from the development of a layered system of supports for a range of mental health conditions, delivered by trained and supervised providers with a defined range of well-defined responsibilities and direct support from qualified managers and support staff. Community outreach should employ a diverse range of strategies by developing advocacy partnerships with the service user and community groups, and engaging community leaders wherever possible. Coordination between program staff, government representatives, and communities is critical to developing clear expectations, continually adjusting the program to better meet changing needs, and maximizing the potential for sustainability. Ongoing collaboration and advocacy with the government is crucial to promoting the adequate investment of funds and human resources to build high-quality, sustainable mental health systems. Future programs can benefit from the lessons learned in this and other successful programs to effectively integrate mental health care within primary health services, making care more accessible to people in need following humanitarian emergencies.

## Footnotes

1 A district with no formerly existing integrated mental health services.

2 A total of 626 community members (48.05% female *v.* 51.95% male) from the surrounding areas received PFA training.

3 Further detail on all quantitative outcome tools is provided in Supplement 2.

4 The lack of a statistically significant finding in this group is likely due to the small sample size as only 17 PSCs completed this test.

5 This data was originally recorded by hand by HBCWs in the field, and there were occasional errors and incidents where the writing was illegible, resulting in missing data. For example, unique identifier IDs were sometimes incorrectly recorded or unverifiable due to human error. For this reason, it was not possible to link the presenting problem data with the outcome data for 52 service users.

## Supplementary material

For supplementary material accompanying this paper visit http://dx.doi.org/10.1017/gmh.2021.8. click here to view supplementary material
