# Supplementary material for: Mental health integration in primary health services after the earthquake in Nepal: a mixed-methods program evaluation
Source: Glob Ment Health (Camb). 2021 Mar 15;8:e10. doi: 10.1017/gmh.2021.8 (PMC8127643; doi:10.1017/gmh.2021.8)
Supplement: Supplementary file 1 [file S205442512100008Xsup001.docx]

**Supplement 1: Overview of Training Packages**

| **Trainee group** | **Aim** | **Curriculum summary** | **Duration of initial training (days)** | **Supervision arrangements** | | **Refresher training** | |
| --- | --- | --- | --- | --- | --- | --- | --- |
| **WHO mhGAP Training Package for Prescribers*** | Equip trainees to identify mental health and psychosocial disorders and provide appropriate treatment. | mhGAP training focused on clinical management (including pharmacological management) of depression, psychosis, epilepsy, Alcohol Use Disorders (AUD), Suicide, PTSD. | 9 | Group supervision by partner NGO psychiatrist (monthly in Dhading, bimonthly in Gorkha & Sindhuli) | | 3 days, 5-6 months after initial training | |
| **WHO mhGAP Training Package for Non-Prescribers** | Equip trainees to identify mental health and psychosocial problems and provide psychosocial support. | mhGAP training focused on non-pharmacological management of depression, psychosis, epilepsy, Alcohol Use Disorders, Suicide, PTSD. | 5 | Monthly group supervision by partner NGO Clinical Psychologists, Senior Counselors, or Health Assistants | | 3 days, 5-6 months after initial training | |
| **Advanced Training for Psychosocial Counselors (PSCs)** | Enhance the skills of practicing PSCs in mental health service provision | The curriculum was designed by TPO and endorsed by the National Health Training Center. Topics included counseling for alcohol problems, family counseling, healthy activities, problem solving, self-help group formation, and providing support to HBCWs. | 6 in Dhading, 12 in Gorkha & Sindhuli** | Monthly group supervision by clinical psychologists or senior counselors | | 5 days in Dhading, 12 days in Gorkha & Sindhuli, 6 months after initial training | |
| **Basic Psychosocial Support Training Package for Home-Based Care Workers** | Develop community members’ skills in basic psychosocial care and appropriate referral. | The curriculum was designed by TPO. Topics included: an introduction to mental health; communication skills; psycho-education; challenging stigma and discrimination; home-based care; and recognition and referral of depression, suicide, AUD, epilepsy, PTSD, psychosis. | 5 in Dhading, 20 in Gorkha & Sindhuli | Monthly group supervision by psychologist/ counselor | | 5 days, 5-6 months after initial training in Dhading only | |
| **Training to upgrade HBCWs to Psychosocial Counselors** | Train HBCWs as government-certified PSCs. | The curriculum was developed by TPO and endorsed by the CTEVT. Topics included anxiety disorders, psychosis, bipolar disorder, developmental delay, suicide, depression, harmful drinking, psychosomatic complaints, trauma, resilience and coping strategies, communication skills, counseling, case management, stress management, stigma and discrimination, clinic management and documentation. | 45 days of classroom-based training and 78 days of supervised field placements | | Monthly group and individual supervision sessions for three months | | NA |
| **FCHV Training Package** | Equip FCHVs with the skills to identify psychosocial problems and mental disorders, and to refer appropriately. | The curriculum was designed by TPO and endorsed by the National Health Training Center. The training focused on the use of the Community Informant Detection Tool (CIDT), a step-by-step guide to identifying priority mental health conditions, and referring to health facilities as appropriate. | 2 | | Monthly supervision by PSCs | | 1 day, 5-6 months after initial training |
| **Mental Health Orientation for Community Leaders** | Increase community leaders’ knowledge about available MHPSS services and equip them to recognize MHPSS needs and refer as appropriate. | The curriculum was designed by TPO. Topics included basic introduction to MHPSS, recognizing mental health symptoms, introduction to counseling, and referral mechanisms. | 1 | | NA | | NA |
| **Psychological First Aid Training** | Equip community leaders to provide emotional and practical help to people who have recently been exposed to a traumatic event. | The curriculum was based on the Psychological First Aid Guide for Field Workers developed by the WHO, the War Trauma Foundation and World Vision. Topics included helping responsibly, principles of PFA, and self-care.† | 1 | | NA | | NA |
| * Training material on PTSD was sourced from the mhGAP Humanitarian Intervention Guide. Material on all other disorders was sourced from the standard mhGAP Intervention guide.  ** The duration of some training courses varied in the different districts due to differences in the budgets of each of the implementing partners.  † A 3-day PFA ‘Training of Trainers’ course was also conducted with participants from a range of governmental bodies and NGOs from 10 different districts. | | | | | | | |

**Supplement 2: Details of quantitative outcome tools**

| **Trainee Outcome Tools**  (Developer) | **Description** | **Respondents** | **First assessment (T1)** | **Last assessment (T2)** |
| --- | --- | --- | --- | --- |
| **Pre- and post-training**  **knowledge tests**  (*TPO)* | Multiple choice knowledge tests corresponding to each training package. | - Prescribers  - Non-prescribers  - PSCs  - HBCWs | First day of training | Last day of training |
| **Perceived Competency**  **Checklist (PCC)**  (IMC) | Questionnaire measuring self-reported perceived competency on two subscales: general skills (e.g. knowledge of the effects of stress on mental health, ability to communicate appropriately with service users) and clinical skills (e.g. e.g. ability to conduct appropriate assessments, diagnoses, interventions and referrals). | - Prescribers  - Non-prescribers  - PSCs | First day of training | Last day of refresher  training |
| **Assessed Clinical**  **Encounter Checklist (ACE)**  (IMC) | Checklist completed by supervisor while observing trainee role-playing a clinical consultation. Evaluates clinical assessment, care planning and record-keeping. | - Prescribers  - Non-prescribers  - PSCs | Within first 3 months  post-training | During refresher  training/ final  supervision |
| **ENhancing Assessment of Common Therapeutic**  **Factors (ENACT-18)** (Kohrt et al., 2015) | Form completed by supervisor while observing trainee role-playing a consultation. Evaluates verbal and non-verbal communication, client-centered discussion, and promotion of realistic  hope. | - Prescribers  - Non-prescribers  - PSCs  - HBCWs | Last day of training | Last day of refresher  training |
| **WHO Disability**  **Assessment Schedule II**  (WHODAS II; WHO) | Standardized tool developed to measure level of disability. It covers six domains of daily functioning: cognition, mobility, self-care, “getting along”, life activities and participation. | Service users receiving care from prescribers | Three months after initial medication prescription | Nine months after  initial medication  prescription |

**Supplement 3: Qualitative thematic framework**

| **Global theme** | **Organizing theme** | **Sub-themes (where applicable)** |
| --- | --- | --- |
| **Effectiveness: Objective 1.** Capacity Building | Impact of training | - Enhanced knowledge and skills - New confidence in detection, diagnosis and treatment - Limited or no prior understanding |
|  | Impact of supervision and refresher trainings | - Supervision as a key strength of the program - Revision and reminders - Putting training into practice - Continued learning - Seeking expert guidance and sharing ideas |
|  | Training feedback and suggestions for improvement | - Simplifying concepts - Use of evidence-based guidelines - Need for better coordination of supervision sessions - Need for more regular supervision and refresher training - Need to cover more disorders and topics - Need to dedicate more time to counseling - Need to separate professional groups |
| **Effectiveness: Objective 2.** Service Provision | Impact on service users | - Hope & understanding that mental illness is treatable - Recovery and symptom reduction - Daily functioning and social interaction - Family support - Livelihood - Finances |
|  | Impact on Health Workers | - Job satisfaction, sense of achievement, feeling appreciated & proud - Professional development - Self-care and wellbeing/personal impact |
|  | Good practices | - Value of home-visits and follow-up - Effective integration and coordination between health workers |
|  | Remaining challenges | - Underutilization of certain roles - No private counseling space - Community suspicion or skepticism - Medication supply issues and mismatch between supply and demand - Problem with staff turn-over |
|  | Suggestions for improvement | - Review medication - Greater involvement of community leaders and traditional healers - Wider geographical remit |
| **Effectiveness: Objective 3.** Awareness and Access | Impact on health worker attitudes | - New interest in psychiatry - New sense of ownership and responsibility for mental health - Reduction in use of stigmatizing language - Reduction in fear of mental health service users - Increase in time spent with service users - Improved communication style |
|  | Impact on community attitudes | - Reduction in stigma - Change in language used to describe people with mental health problems - New understanding of causes and treatments for mental health problems - Limited impact – more time and awareness required |
|  | Impact on service user access | - New confidence in revealing mental health problems - Increased access |
|  | Recommendations: Awareness and Access | - Awareness activities in all VDCs - Street plays, dramas and art exhibitions - Greater involvement of community leaders |
| **Structural impact** | Impact on policy | - District-level procurement of psychotropic medications - Revisions to the Free Essential Drug List of Nepal - Revisions to National Standard Treatment Protocols |
|  | Social impact | - Generating Demand - Reduction in social problems (e.g. gender-based violence) |
|  | Improved data and documentation |  |
| **Relevance** | Pertinent after earthquake | - Emergency as impetus for change |
|  | Appropriateness of services | - Needs of SUs well met |
|  | Appropriateness of training | - Effective compared with other programs |
|  | Remaining unmet needs | - Need to train a greater number of staff - Need to cover more geographical areas |
| **Sustainability** | Continuation of services | - Mixed views about whether government-affiliated staff will continue to provide MHPSS services - Mixed views about whether partner-affiliated community-based staff will continue to provide services - Medication supply |
|  | Perception that program too short for sustainability | - Changing attitudes takes time - Government buy-in takes time - Improvements in mental health take time - Sense that program “ending in the middle” |
